# Development and Evaluation of Novel Self-Nanoemulsifying Drug Delivery Systems Based on a Homolipid from *Capra hircus* and Its Admixtures with Melon Oil for the Delivery of Indomethacin

**DOI:** 10.1155/2014/340486

**Published:** 2014-03-20

**Authors:** Nicholas C. Obitte, Kenneth C. Ofokansi, Franklin C. Kenechukwu

**Affiliations:** ^1^Department of Pharmaceutical Technology and Industrial Pharmacy, Faculty of Pharmaceutical Sciences, University of Nigeria, Nsukka 410001, Nigeria; ^2^Drug Delivery Research Unit, Department of Pharmaceutics, Faculty of Pharmaceutical Sciences, University of Nigeria, Nsukka 410001, Nigeria

## Abstract

In this study, goat fat (*Capra hircus*) and melon oil were extracted and used to formulate self-nanoemulsifying drug delivery systems (SNEDDS) based on either goat fat alone or its admixture with melon oil by employing escalating ratios of oil(s), surfactant blend (1 : 1 Tween 60 and Tween 80), and cosurfactant (Span 85), with or without carbosil, a glidant, for the delivery of indomethacin. The formulations were encapsulated in hard gelatin capsules and then assessed using isotropicity test, aqueous dilution stability and precipitation propensity, absolute drug content, emulsification time, *in vitro* drug release, and anti-inflammatory activity. The SNEDDS exhibited low precipitation propensity and excellent stability on copious dilution, as well as high drug release *in vitro* and * in vivo*. The inhibition produced by the SNEDDS was comparable to that of indomethacin injection (positive control) for much of the 5 h test period, indicating a high degree of bioavailability of the administered SNEDDS. The absolute drug contents and emulsification times fell within narrow limits. This study has shown that a 1 : 1 ratio of melon oil and goat fat could confer favourable properties with respect to drug release and anti-inflammatory activity on SNEDDS for the delivery of indomethacin, thus encouraging further development of the formulations.

## 1. Introduction

The nonsteroidal anti-inflammatory drugs (NSAIDs) have remained the mainstay of the treatment and management of inflammatory disorders. Despite enormous innovations in novel drug delivery systems (NDDS) through alternative routes, oral drug delivery of NSAIDs has been the most common and preferred route of drug administration [1]. Its status is primarily a consequence of the wide acceptability of this “natural” route, better safety vis-à-vis the parenteral route, low cost of therapy, and improved patient compliance. With an increasing number of lipophilic drugs under development, homolipids and heterolipids have gained renewed interests as excipients for myriads of drug delivery systems [2]. Lipid-based formulations have been shown to enhance the bioavailability of orally administered drugs [3–6]. The oral delivery of lipophilic drugs presents a major challenge because of the low aqueous solubility. The Biopharmaceutics classification system (BCS) [7] classifies drugs into four categories depending on their solubility and permeability characteristics. According to this scheme, indomethacin belongs to class II drugs whose solubility is too low to be consistent with complete absorption. For this class of compounds, defined as “low solubility/high permeability class,” dissolution in the lumen environment is the rate-controlling step in the absorption process [7]. Various formulation approaches have been employed to enhance the oral bioavailability of lipophilic drugs to increase their clinical efficacy, including the use of self-emulsifying drug delivery systems (SEDDS).

Self-emulsifying drug delivery systems (SEDDS) refer to a mixture of oil, surfactant, and cosurfactant, which are capable of fast self-emulsification in the gastrointestinal fluid under mild agitation provided by gastrointestinal motility to form emulsion. The lipid emulsions have advantages in terms of high drug loading capacity, reduction in irritation or toxicity of the incorporated drug, the possibility of sustained release and industrial productivity, improved dissolution of the drug in the GIT [8–13], reduction of presystemic as well as systemic clearance of drug [14, 15], and the possibility of dose reduction. They are thus considered appropriate drug carriers for highly lipophilic drugs. Self-emulsifying drug delivery systems appear to be favoured because of short processing steps and also cost effectiveness due to reliance on cheap raw materials. It is also amenable to scale-up. SEDDS also produce more reproducible plasma concentrations of drugs [16]. The behaviour of such systems can be modified by the use of oil and surfactants in different ratios and by manipulating the polarity and charge of the dispersed globules. Preferred oily components are the medium chain triglycerides, which are, however, limited by low solvent capacity for surfactants or drugs. Semisynthetic medium chain triglycerides having amphiphilic behaviour may also be utilized, with better solvent effects.

Against the above background, a self-nanoemulsifying drug delivery system (SNEDDS) was formulated with a view to surmounting some of the problems encountered when indomethacin is given either orally or parenterally, since anti-inflammatory activity relies on improved bioavailability and quick attainment of reproducible plasma concentrations of the drug [17]. With some disturbing side effects of indomethacin like incidence of GIT irritation following oral administration and difficulties encountered with a rather invasive parenteral administration of indomethacin, a convenient oral dosage form with improved oral bioavailability, reduced doses, and improved anti-inflammatory efficacy was needed to replace conventional oral administration of indomethacin or compliment parenteral therapy involving indomethacin in patients. The SNEDDS would form a nanoemulsion with rapid gastric emptying time and with a wide absorption surface for drug that minimizes prolonged local contact effects [17]. A previous report by our research team indicates that SNEDDS based on melon oil and its admixtures with a homolipid from tallow fat (*Bos indicus*) could be employed as a novel carrier for improved delivery of indomethacin [18]. SNEDDS based on homolipids from other cheap and commonly available fats such as goat fat (*Capra hircus*), dika fat (*Irvingia gabonensis*), and beeswax could also be investigated as novel carriers for improved delivery of indomethacin.

Consequently, the objective of this study was, therefore, to investigate the suitability of a homolipid from* Capra hircus* or its admixture with melon oil, for use as the oily component for indomethacin-loaded SNEDDS, using standard nontoxic surfactants. These lipids are of low cost and readily available in Sub-Saharan Africa. A homolipid from* Capra hircus* has previously been evaluated as a lipid matrix for suppositories [19]. It is expected that the formulations would not only show enhanced bioavailability but also better tolerance due to nontoxicity of edible oils.

## 2. Materials and Methods

### 2.1. Materials

Goat (*Capra hircus*) fat was obtained from the Nsukka abattoir while melon seeds were procured locally from Nsukka Main Market, Enugu State, Nigeria. Indomethacin powder (Medrel Pharmaceuticals, Pvt Ltd, India), Tween 65, Tween 80, and Span 85 (Merck, Damstadt, Germany) were also used in the study. All other reagents were of analytical grade and used without further modification.

### 2.2. Extraction of Melon Oil

The melon seeds were dried, milled, and then extracted by cold maceration for 24 h using petroleum ether (20–40°C boiling point). The resulting oil was bleached by treating with activated charcoal at 80–90°C for 1 h. Thereafter, the oil was recovered under reduced pressure (vacuum) and at low temperature using a rotary evaporator.

### 2.3. Extraction of Homolipid from* Capra hircus*

About 1 kg of* Capra hircus* homolipid (goat fat) was processed in the laboratory by the process of rendering as reported previously [19]. This was followed by straining using a porcelain cloth. After cooling, the homolipid was recovered by simple decantation of the lower aqueous layer.

### 2.4. Proximate Analysis

Percent concentrations of proteins, lipid, carbohydrate, crude fibre, moisture, and ash in melon oil and the homolipid from goat fat were determined using standard procedures [20].

### 2.5. Purification of Melon Oil

Activated charcoal, at a concentration of 2% w/w, was employed in purifying the oil, by heating the dispersion of charcoal and oil/fat at 80–90°C for 1 h, followed by vacuum filtration using a Buchner funnel.

### 2.6. Preformulation Isotropicity Test

Various batches of SNEDDS were prepared based on escalating ratios of melon oil, goat fat, surfactants, and cosurfactant. The preparation was achieved by simple mixing of weighed components in a beaker over a water bath at 45–50°C for 15 min. After storage of the resulting dispersions at ambient conditions for 24 h, visual examination was conducted for evidence of phase separation [21]. Only ratios which remained isotropic after this storage time were used in the formulation of SNEDDS.

### 2.7. Formulation of SNEDDS and Encapsulation

The preparation of SNEDDS batches was done according to the proportions depicted in Tables 1 and 2. In each case, weighed amounts of oil(s), surfactants, cosurfactant, and indomethacin (20 mg), with or without carbosil (15 mg), were mixed manually with the aid of a stirring rod for 10 min in a beaker over a water bath at 50°C. Carbosil, a glidant, is believed to promote dispersion behaviour. Encapsulation of the SNEDDS from the different batches was carried out by transferring a mass containing exactly 20 mg of indomethacin into a 450 mg capacity hard gelatin capsule. Calculations for each batch of the indomethacin-loaded SNEDDS were done to give twenty capsules from which amounts containing exactly 20 mg indomethacin were weighed and encapsulated.

### 2.8. Photon Correlation Spectroscopy (PCS)

Submicron particle size analysis was performed using a Zetasizer nano (ZEN 3600, Malvern Instruments, UK). Measurements were made at 25°C at a scattering angle of 90°. The mean particle size and polydispersity index were determined in a single run while the zeta potential was similarly determined by phase analysis light scattering (PALS) using the same instrument.

### 2.9. Postformulation Isotropicity Test

Indomethacin-loaded SNEDDS were allowed to stand for 24 h and then visually examined for phase separation [22] to identify stable preparations. All successful batches were encapsulated into 20 unit doses by enclosing a mass of product equivalent to 20 mg indomethacin in a 450 mg capacity hard gelatin capsule.

### 2.10. Dilution Stability and Precipitation Propensity Test

One capsule from each batch was discharged into 100 mL of 0.1 N HCl. The resulting solution was transferred to a beaker and diluted with successive 100 mL volumes until the 1 litre mark was reached. The system was allowed to stand for 2 h and then checked for drug precipitation or phase separation. From this diluted solution, a 10 mL volume was withdrawn, transferred into a test tube, and securely covered. It was then allowed to stand for 24 h and visually inspected again for signs of drug precipitation.

### 2.11. Determination of Emulsification Time

A capsule from each batch was emptied into a 100 mL beaker containing 0.1 M HCl. The beaker was mounted on a magnetic stirrer hot plate assembly and stirred at 50 rpm and 37 ± 1°C until complete emulsification occurred, as indicated by constant turbidity. A mean of triplicate determinations was taken as the emulsification time for each batch.

### 2.12. Absolute Drug Content

A calibration curve for indomethacin in alcohol was obtained by diluting a 2 mg% alcoholic solution of indomethacin serially with the solvent to obtain several dilute concentrations ranging between 0.1 mg% and 1 mg%. The absorbance of each concentration was determined at a predetermined wavelength of 232 nm, against a blank consisting of alcohol, using a spectrophotometer (Phoenix-220 DPC V model). For determination of the absolute drug content, a capsule from each batch was emptied into a 250 mL beaker and emulsified by the addition of about 90 mL of 0.1 N HCl. The resulting solution was made up to 100 mL with 0.1 N HCl. A 0.1 mL volume was withdrawn and diluted to 10 mL with alcohol. The absorbance of the resulting solution was then determined, against a blank consisting of a mixture of solutions of 0.1 N HCl and alcohol, with a spectrophotometer, and the amount of indomethacin calculated from a calibration plot previously determined for indomethacin. Five replicate experiments were done and the mean of five determinations was taken to be the absolute drug content for each batch.

### 2.13. *In Vitro* Release Studies

*In vitro *release study was performed using USP XXII rotating paddle apparatus (Erweka, Germany). The dissolution medium consisted of 900 mL of freshly prepared 0.1 N HCl (pH 1.2) maintained at 37 ± 0.5°C. The polycarbonate dialysis membrane used was pretreated by soaking it in the dissolution medium for 24 h prior to the commencement of each release experiment. A capsule from each batch was enclosed in a dialyzing membrane (6 cm length × 3 cm width) containing 2 mL of the dissolution medium, which was tied at both ends and introduced into a dissolution apparatus containing 900 mL of 0.1 N HCl. Mild agitation was provided at a speed of 50 rpm at 37 ± 0.5°C. At predetermined time intervals, 5 mL volumes were withdrawn, filtered, and assayed spectrophotometrically for indomethacin after appropriate dilution. The release medium was replenished after each withdrawal to maintain constant volume.

### 2.14. *In Vivo* Anti-Inflammatory Studies

The study was conducted in accordance with Ethical Guidelines of Animal Care and Use Committee (Research Ethics Committee) of the University of Nigeria, Nsukka, following the Federation of European Laboratory Animal Science Association and the European Community Council Directive of November 24, 1986 (86/609/EEC) [23]. Female Wistar rats (150–250 g) were housed in cages under controlled temperature and humidity and under a photoperiod schedule of 12 h light/12 h dark. They were fed a standard laboratory animal diet and tap water was provided* ad libitum*. The rats had free access to food and water prior to the commencement and throughout the duration of the experiment in order to mitigate the gastroerosive side effects of the administered loaded drug. The animals were divided into four groups of five rats each. A 50% aqueous dispersion of egg albumin was used as the phlogistic agent. Group 1 animals received, intraperitoneally, 0.357 mg of indomethacin injection per kg body weight of rat and served as the positive control, while group 4 animals, serving as negative control, received a volume (437 *μ*L) of freshly distilled water equal to the volume of the administered injection. Groups 2 and 3 received, orally, 437 *μ*L each of SNEDDS 2A and SNEDDS 4C, respectively, with the help of an intragastric tube. One hour after initial drug administration in each case, 50 *μ*L of the phlogistic agent was injected into the subplantar surface of the right hind paw. Oedema was assessed based on the difference between linear circumference (*C*_*o*_) of the injected paw at time zero and the circumference (*C*_*t*_) after time, *t*. Percentage inflammation (and hence inhibition) was calculated using the following relationship:

(1) $$\begin{array}{c} \text{Inflammation}\;\left(\%\right)=\frac{{\text{AI}}_{t}}{{\text{AI}}_{c}}\times 100, \end{array}$$

where AI_*t*_ is the average inflammation at time, *t*, and AI_*c*_ is the average inflammation of control animals at the same interval. From the values obtained, percentage inhibition (100% minus percentage inflammation) was calculated.

### 2.15. Statistical Analysis

Statistical analysis was performed using SPSS statistical package. Mean and standard error for all data were calculated. For batch comparisons, the analysis of variance (ANOVA) was used to determine statistically significant differences at *P* < 0.05.

## 3. Results and Discussion

### 3.1. Proximate Analysis, Dilution Stability, and Precipitation Propensity Test of the SNEDDS

The development of self-emulsifying drug delivery systems depends on the use of modified vegetable oils [24], vegetable oils, or semisynthetic medium chain triglycerides with amphiphilic nature [25, 26]. Blending of triglycerides with mono- and diglycerides [27] or with semisynthetic medium chain triglycerides that have amphiphilic character enhances the inherently poor solvent properties of triglycerides. Preliminary results of the proximate analysis of melon seed oil, edible oil consumed widely in Nigeria, revealed that the oil is predominantly composed of fat, mostly triglycerides (93.8%), while ash and fiber were present in trace amounts. The amount of protein was 0.87% while moisture constituted 0.2% of the oil. Preliminary phase separation studies were initially carried out as a preliminary test for the efficiency of the self-nanoemulsification between the surfactant and the lipidic blends. The formulations tested were isotropic and none showed any evidence of phase separation. Moreover, all batches of the formulated SNEDDS retained their isotropicity after a tenfold dilution. There were no signs of drug precipitation on visual inspection after dilution.

The medium term stability of the formulations was assessed by storing them at room temperature (28°C) on a bench for four weeks. No phase separation or change in the integrity of the formulation was observed. This indicates a high level of stability of goat fat-based SNEDDS. The principal characteristic of self-emulsifying systems is their ability to form fine o/w microemulsions or nanoemulsions spontaneously upon mild agitation following dilution by aqueous phases [8–15]. This is a result of thermodynamic stability of self-emulsifying systems as opposed to the regular emulsions that are thermodynamically unstable. Incorporation of indomethacin did not affect the stability of the formulation throughout the duration of the experiment. In other words, the SNEDDS formulated from blends of a homolipid from* Capra hircus* remained isotropic after drug loading. This uniform structure is necessary to avoid postformulation drug partitioning upon phase separation [28]. Based on this assessment alone, preference should be given to ratios with lower surfactant concentrations to avoid adverse effects due to use of surfactant at high concentrations. All batches showed high stability on copious dilution with aqueous fluid, as well as a low tendency to precipitate the loaded drug. This is due to the high thermodynamic stability of the resulting oil-in-water nanoemulsion. The low precipitation propensity is a valuable property of such systems, in which deposition of drug may not be expected to occur due to pH change or dilution in the internal environment of the body. This should minimise dose dumping. However, extremely low propensity to deposit drug may limit efficacy, since activity is normally due to free drug and not the reservoir fraction.

SNEDDS are reputed for high stability due to formation of thermodynamically inert microemulsions on contact with water under gentle agitation [29, 30]. The solubility of hydrophobic drugs and therefore their bioavailability are improved in such systems by selective dissolution/partitioning in the oily component [31]. Particle sizes in such systems are of the order of 100 nm or less [9, 11, 32]. Stability from precipitation on dilution is a critical design parameter which depends on the solubility of drug in various formulation components [9], particularly in the oil. The capacity of the oil to dissolve and hold the hydrophobic drug is improved in the presence of surfactants and hence the use of Span 85, a lipophile, as cosurfactant.

### 3.2. Determination of Emulsification Time

The mean emulsification times for triplicate determinations obtained for SNEDDS formulations are presented in Table 3. All of the samples became completely emulsified within 4 min under the temperature and stirring conditions employed in the experiment. The mean emulsification time obtained for SNEDDS formulated with the homolipid alone ranged from 0.44 ± 0.15 to 1.54 ± 0.22 min while formulations containing an admixture of melon oil with the homolipid had average emulsification times which ranged between 0.58 ± 0.08 and 1.85 ± 0.14 min. Carbosil-containing batches took slightly longer times to emulsify. In other words, the incorporation of carbosil into the goat fat-based formulations resulted in the prolongation of the time for complete emulsification to between 2.35 ± 0.23 and 3.13 ± 0.20 min while formulations containing melon oil together with the homolipid from* Capra hircus* and carbosil had a mean emulsification time range of 3.33 ± 0.25 to 3.79 ± 0.34 min. It is discernible from Table 3 that the inclusion of carbosil markedly affected the mean emulsification time. Emulsification rate is an important parameter in emulsification efficiency [21] and therefore product performance. In these cases, inclusion of carbosil increased the emulsification times beyond the 2 min time recommended for such systems [33]. Rapid self-emulsification occurs when the entropy change that favours dispersion of the SNEDDS is greater than the work required for increasing the surface area during dispersion [34]. Such free energy change should either be low, but positive, or negative [35]. The effect of carbosil is a direct consequence of its viscosity imparting effect.

### 3.3. Absolute Drug Content

The absolute drug content of all the batches tested is presented in Table 4. The absolute drug contents of the formulated batches of SNEDDS did not show any remarkable deviation from the theoretical content and ranged between 15.18 ± 1.07 and 19.60 ± 1.58 mg of indomethacin. The fact that drug contents of the batches varied between narrow limits (low standard deviation) is an indication of drug solubility in the SNEDDS base, otherwise sedimentation influences would have caused drug settling and marked variation in drug content between batches. This process yields uniform drug contents and is therefore amenable to scale-up. Moreover, reproducibility is a common feature of self-emulsifying drug delivery systems (SEDDS), due to uniform drug loading and dispersion. The formulated SNEDDS gave reproducible absolute drug contents not varying much between batches, consistent with previous study on SNEDDS [36].

### 3.4. Droplet Size and Zeta Potential

**Figure 1:**
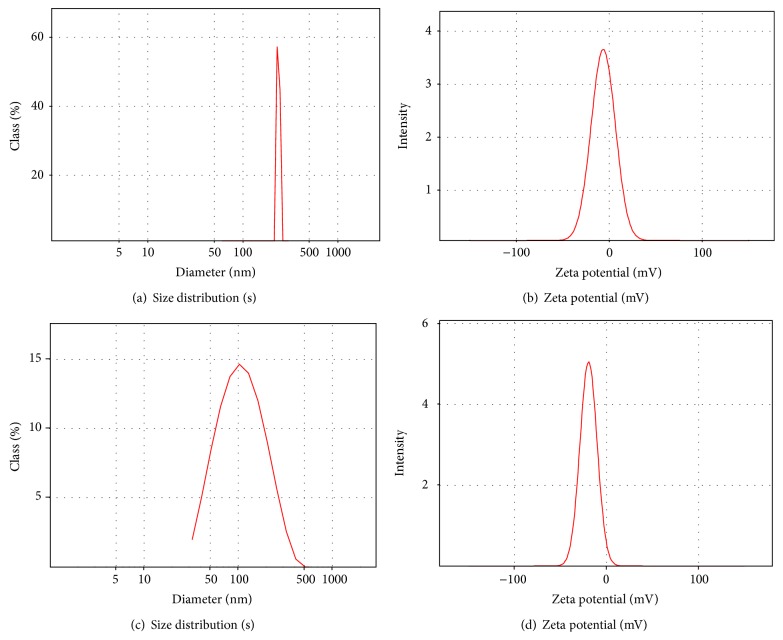
Particle size distribution and zeta potential of the SNEDDS as measured by photon correlation spectroscopy. Mean size of goat fat only-based SNEDDS (a), zeta potential of goat fat only-based SNEDDS (b), mean size of goat fat/melon oil blend-based SNEDDS (c), and zeta potential of goat fat/melon oil blend-based SNEDDS (d).

The mean diameters and zeta potentials of the SNEDDS as measured by photon correlation spectroscopy (PCS) are shown in Figures 1(a)–1(d). The mean diameter of the SNEDDS was 195 nm for goat fat only-based SNEDDS with a polydispersity index of 0.25 indicating monodisperse size distribution and 353 nm for SNEDDS based on admixture of goat fat and melon oil with a polydispersity index of 0.0665 depicting also a unimodal size distribution having a somewhat broader particle size distribution when compared with SNEDDS formulated with goat fat only as shown in Figures 1(a) and 1(c). Similarly, goat fat only-based SNEDDS had a zeta potential of −7.2 mV while that of melon oil/goat fat-based SNEDDS was −16.4 mV as shown in Figures 1(b) and 1(d). Generally, particle sizes in SNEDDS are in the nanometer size range [9, 32]. Measurement of the droplet size could be done by photon correlation spectroscopy after extensive dilution [11]. In general, the stability of a SNEDDS depends upon the particle size, emulsion droplet charge, and droplet polarity. These influences are in turn governed by the nature (HLB, chain length, and degree of unsaturation) and concentration of the surfactant employed.

### 3.5. *In Vitro* Release Studies

**Figure 2:**
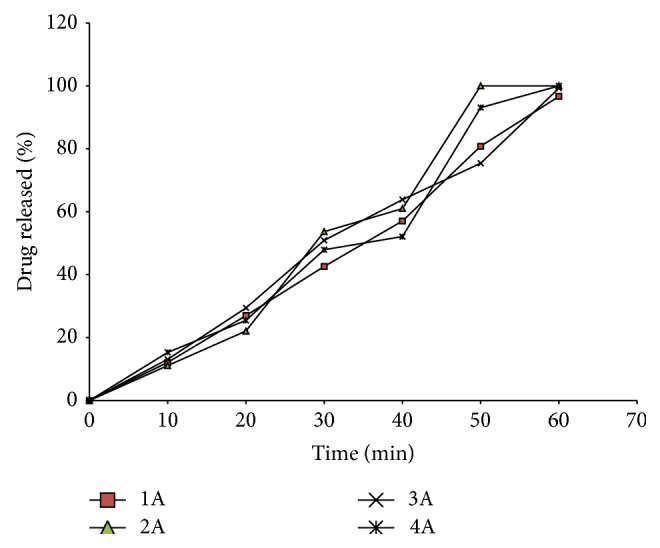
Release profile of indomethacin from SNEDDS based on goat fat combined in different ratios with a surfactant blend (1 : 1 Tween 60 and 80 blend) and cosurfactant (Span 85).

**Figure 3:**
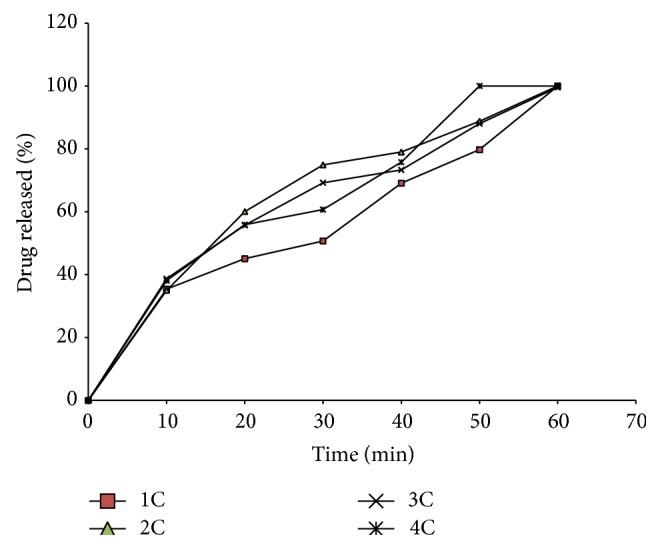
Release profiles of indomethacin from SNEDDS based on goat fat and melon oil blend (1 : 1) combined in different ratios with a surfactant blend (1 : 1 Tween 60 and Tween 80 blend) and cosurfactant (Span 85).

The release properties of the formulated SNEDDS are shown in Figures 2 and 3. All batches of the SNEDDS tended to delay the release of drug beyond 40 min. This could be of special benefit especially in drugs with gastric mucosal toxicity such as indomethacin.

However, the 40 min lag is too short, and it is likely that release of product would still occur in the stomach since most materials have residence times longer than 40 min in the stomach. But then SNEDDS are known to have short residence times due to the ultrafine particle size of the resulting micro/nanoemulsion which promotes gastric emptying [21]. In goat fat only-based SNEDDS (1A, 1B, 2A, 2B, 3A, 3B, 4A, and 4B), drug release from the various batches (Figure 2) was marginal within the first 20 min but increased gradually thereafter attaining 96.65, 99.30, and 100% release at 60 min for batches 1A, 3A, and 4A, respectively. However, batch 2A, formulated from goat fat, surfactants, and cosurfactants in the ratio 35 : 45 : 20, recorded 100% release at 50 min, implying that the most extended release formulation could be obtained from this batch. Interestingly, SNEDDS formulated with an admixture of melon oil and the studied homolipid exhibited a biphasic release pattern (Figure 3) characterized by a rapid initial release of more than 35% of indomethacin within the first 10 min, followed thereafter by a more gradual drug release up to the 50th min, such that almost 100% of the drug was released in 1 h, but batch 4C, formulated from goat fat/melon oil blend (1 : 1), surfactants, and cosurfactants in the ratio 25 : 60 : 15, recorded 100% drug release at 50 min. Drug release from self-emulsifying systems (SES) is dependent on droplet size and polarity, which in turn depends on the nature and concentration of surfactants and cosurfactant, as well as on the degree of unsaturation of the lipid.

### 3.6. *In Vivo* Anti-Inflammatory Studies

**Figure 4:**
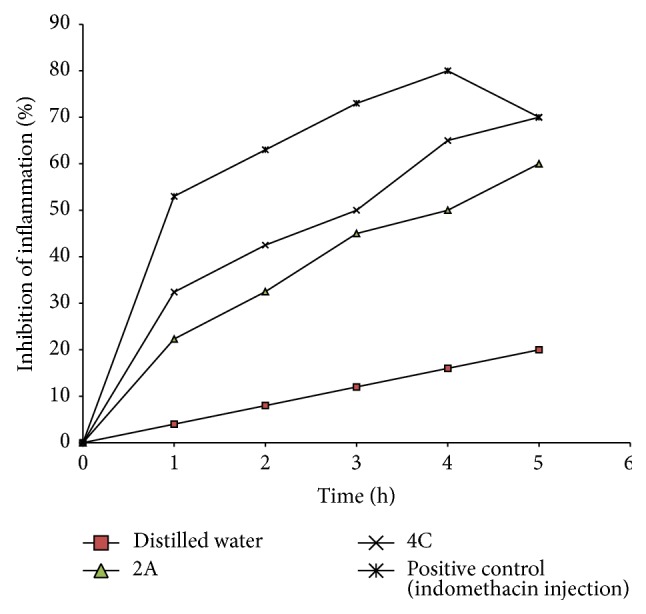
Inhibition of egg albumin-induced inflammaution by indomethacin contained in self-emulsifying drug delivery systems. Note that 2A contains indomethacin in SNEDDS formulated from goat fat with surfactant blend (1 : 1 of Tween 60 and Tween 80) and cosurfactant (Span 85) in the ratio of 35 : 45 : 20, respectively; 4C contains indomethacin in SNEDDS formulated from goat fat and melon oil blend (1 : 1) combined with surfactant blend (1 : 1 of Tween 60 and Tween 80) and cosurfactant (Span 85) in the ratio of 25 : 60 : 15, respectively.

The* in vivo *anti-inflammatory properties of the formulations administered are shown in Figure 4 as a function of time. The administered SNEDDS reduced the degree of inflammation due to the phlogistic agent. Indomethacin inhibits inflammation by antagonizing the cyclooxygenase enzyme required for prostaglandins synthesis [17]. The inhibition produced by the positive control and the drug-loaded SNEDDS was identical for much of the 5 h test period indicating a high degree of bioavailability of the administered SNEDDS. Goat fat/melon oil blend-based SNEDDS achieved 70% inhibition of inflammation within the 5 h test period while SNEDDS based solely on goat fat equally inhibited inflammation but to a lesser degree (55%) when compared to that produced by goat fat/melon oil-based systems.

## 4. Conclusion

In this study, formulation of indomethacin as SNEDDS not only preserved the activity of the drug but also guaranteed an anti-inflammatory activity comparable to that of indomethacin injection indicating a high degree of bioavailability of the administered SNEDDS. This is especially true for goat fat/melon oil blend-based SNEDDS which achieved 70% inhibition of inflammation within the 5 h test period. SNEDDS based solely on goat fat equally inhibited inflammation but to a lesser degree (55%) when compared to that produced by goat fat/melon oil-based systems. The observed anti-inflammatory activity of indomethacin-loaded SNEDDS may be due to large absorption surface area brought about by emulsification. This postulated large surface area that facilitated absorption was greater for goat fat/melon-based SNEDDS than for systems based only on goat fat. This study has shown that a 1 : 1 ratio of melon oil and goat fat could confer favourable properties with respect to drug release and anti-inflammatory activity on SNEDDS for the delivery of indomethacin, thus encouraging further development of the formulations.

## Figures and Tables

**Table 1:** Composition of indomethacin-loaded SNEDDS based on goat fat only.

Batch	Ratio∗ O : S : CS	Goat fat (g)	Tween 65 (g)	Tween 80 (g)	Span 85 (g)	Carbosil (mg)	Indomethacin (mg)
1A	20 : 60 : 20	0.8	1.2	1.2	0.8	—	400
1B	20 : 60 : 20	0.8	1.2	1.2	0.8	300	400
2A	35 : 45 : 20	1.4	0.9	0.9	0.8	—	400
2B	35 : 45 : 20	1.4	0.9	0.9	0.8	300	400
3A	25 : 55 : 20	1.0	1.1	1.1	0.8	—	400
3B	25 : 55 : 20	1.0	1.1	1.1	0.8	300	400
4A	25 : 60 : 15	1.0	1.2	1.2	0.8	—	400
4B	25 : 60 : 15	1.0	1.2	1.2	0.8	300	400

^*^Ratio of oil (goat fat) : surfactant blend (Tween 65 and Tween 80) : cosurfactant (Span 85).

**Table 2:** Composition of indomethacin-loaded SNEDDS based on a blend of goat fat and melon oil.

Batch	Ratio∗ O : S : CS	Goat fat (g)	Melon oil (g)	Tween 65 (g)	Tween 80 (g)	Span 85 (g)	Carbosil (mg)	Indomethacin (mg)
1C	20 : 60 : 20	0.4	0.4	1.2	1.2	0.8	—	400
1D	20 : 60 : 20	0.4	0.4	1.2	1.2	0.8	300	400
2C	35 : 45 : 20	0.7	0.7	0.9	0.9	0.8	—	400
2D	35 : 45 : 20	0.7	0.7	0.9	0.9	0.8	300	400
3C	25 : 55 : 20	0.5	0.5	1.1	1.1	0.8	—	400
3D	25 : 55 : 20	0.5	0.5	1.1	1.1	0.8	300	400
4C	25 : 60 : 15	0.5	0.5	1.2	1.2	0.8	—	400
4D	25 : 60 : 15	0.5	0.5	1.2	1.2	0.8	300	400

^*^Ratio of oil blend (goat fat and melon oil) : surfactant blend (Tween 65 and Tween 80) : cosurfactant (Span 85).

**Table 3:** Emulsification time of batches of indomethacin-loaded SNEDDS.

Batch∗	Ratio of oil blend : surfactant blend : cosurfactant∗∗	Mean time ± S.E.M. (min)
1A	20 : 60 : 20	1.54 ± 0.22
1B	20 : 60 : 20	2.89 ± 0.16
1C	20 : 60 : 20	1.85 ± 0.14
1D	20 : 60 : 20	3.52 ± 0.28
2A	35 : 45 : 20	0.44 ± 0.15
2B	35 : 45 : 20	2.35 ± 0.23
2C	35 : 45 : 20	0.58 ± 0.08
2D	35 : 45 : 20	3.63 ± 0.22
3A	25 : 55 : 20	1.03 ± 0.25
3B	25 : 55 : 20	3.13 ± 0.20
3C	25 : 55 : 20	1.18 ± 0.33
3D	25 : 55 : 20	3.33 ± 0.25
4A	25 : 60 : 15	1.30 ± 0.28
4B	25 : 60 : 15	2.86 ± 0.18
4C	25 : 60 : 15	1.54 ± 0.37
4D	25 : 60 : 15	3.79 ± 0.34

^*^A contains goat fat, B contains goat fat and carbosil, C contains goat fat with melon oil, and D contains goat fat with melon oil and carbosil. ∗∗All batches contain lipid (either goat fat or a 1 : 1 blend of goat fat with melon oil), surfactant (1 : 1 blend of Tween 60 and Tween 80), and Span 85 combined in the ratio shown above.

**Table 4:** Absolute drug contents of indomethacin-loaded SNEDDS.

Batch∗	Ratio of oil blend : surfactant blend : cosurfactant	Absolute drug contents (mg)∗∗ ± SD
1A	20 : 60 : 20	15.34 ± 0.96
1B	20 : 60 : 20	16.60 ± 1.28
1C	20 : 60 : 20	16.70 ± 1.09
1D	20 : 60 : 20	19.36 ± 1.83
2A	35 : 45 : 20	15.18 ± 1.07
2B	35 : 45 : 20	17.40 ± 0.84
2C	35 : 45 : 20	18.10 ± 0.53
2D	35 : 45 : 20	19.05 ± 1.91
3A	25 : 55 : 20	17.30 ± 0.82
3B	25 : 55 : 20	18.50 ± 1.65
3C	25 : 55 : 20	15.47 ± 1.26
3D	25 : 55 : 20	19.60 ± 0.99
4A	25 : 60 : 15	18.10 ± 0.74
4B	25 : 60 : 15	19.60 ± 1.58
4C	25 : 60 : 15	19.30 ± 1.92
4D	25 : 60 : 15	18.41 ± 1.87

^*^A contains goat fat, B contains goat fat and carbosil, C contains goat fat with melon oil, and D contains goat fat with melon oil and carbosil. ∗∗All batches were intended to deliver 20 mg of indomethacin.

## Abbreviations

SES: Self-emulsifying systems
SEDDS: Self-emulsifying drug delivery systems
SNEDDS: Self-nanoemulsifying drug delivery systems
BCS: Biopharmaceutics classification system
PALS: Phase analysis light scattering
PCS: Photon correlation spectroscopy
NSAIDs: Nonsteroidal anti-inflammatory agents
NDDS: Novel drug delivery systems
ANOVA: Analysis of variance.

## References

[1] Gupta H., Bhandari D., Sharma A. (2009). Recent trends in oral drug delivery: a review. *Recent Patents on Drug Delivery and Formulation*.

[2] Stuchlík M., Zák S. (2001). Lipid-based vehicle for oral drug delivery. *Biomedical Papers of the Medical Faculty of the University Palacky, Olomouc, Czechoslovakia*.

[3] Hou D., Xie C., Huang K., Zhu C. (2003). The production and characteristics of solid lipid nanoparticles (SLNs). *Biomaterials*.

[4] Sarkar N. N. (2002). Mifepristone: bioavailability, pharmacokinetics and use-effectiveness. *European Journal of Obstetrics Gynecology and Reproductive Biology*.

[5] Gao P., Guyton M. E., Huang T., Bauer J. M., Stefanski K. J., Lu Q. (2004). Enhanced Oral Bioavailability of a Poorly Water Soluble Drug PNU-91325 by Supersaturatable Formulations. *Drug Development and Industrial Pharmacy*.

[6] You J., Cui F.-D., Li Q.-P., Han X., Yu Y.-W., Yang M.-S. (2005). A novel formulation design about water-insoluble oily drug: preparation of zedoary turmeric oil microspheres with self-emulsifying ability and evaluation in rabbits. *International Journal of Pharmaceutics*.

[7] Amidon G. L., Lennernas H., Shah V. P., Crison J. R. (1995). A theoretical basis for a biopharmaceutic drug classification: the correlation of in vitro drug product dissolution and in vivo bioavailability. *Pharmaceutical Research*.

[8] Kumar S., Gupta S. K., Sharma P. K. (2012). Self-emulsifying drug delivery systems (SEDDS) for oral delivery of lipid based formulations- a review. *African Journal of Basic & Applied Science*.

[9] Mistry R. B., Sheth N. S. (2011). A review: self emulsifying drug delivery system. *International Journal of Pharmacy and Pharmaceutical Sciences*.

[10] Wei L., Sun P., Nie S., Pan W. (2005). Preparation and evaluation of SEDDS and SMEDDS containing carvedilol. *Drug Development and Industrial Pharmacy*.

[11] Arida A. I., Al-Tabakha M. M., Hamoury H. A. J. (2007). Improving the high variable bioavailability of griseofulvin by SEDDS. *Chemical and Pharmaceutical Bulletin*.

[12] Holm R., Porter C. J. H., Edwards G. A., Müllertz A., Kristensen H. G., Charman W. N. (2003). Examination of oral absorption and lymphatic transport of halofantrine in a triple-cannulated canine model after administration in self-microemulsifying drug delivery systems (SMEDDS) containing structured triglycerides. *European Journal of Pharmaceutical Sciences*.

[13] Shi S., Chen H., Lin X., Tang X. (2010). Pharmacokinetics, tissue distribution and safety of cinnarizine delivered in lipid emulsion. *International Journal of Pharmaceutics*.

[14] Shen H., Zhong M. (2006). Preparation and evaluation of self-microemulsifying drug delivery systems (SMEDDS) containing atorvastatin. *Journal of Pharmacy and Pharmacology*.

[15] Patel D., Sawant K. K. (2007). Oral bioavailability enhancement of acyclovir by self-microemulsifying drug delivery systems (SMEDDS). *Drug Development and Industrial Pharmacy*.

[16] Hong J.-Y., Kim J.-K., Song Y.-K., Park J.-S., Kim C.-K. (2006). A new self-emulsifying formulation of itraconazole with improved dissolution and oral absorption. *Journal of Controlled Release*.

[17] Kim J. Y., Ku Y. S. (2000). Enhanced absorption of indomethacin after oral or rectal administration of a self-emulsifying system containing indomethacin to rats. *International Journal of Pharmaceutics*.

[18] Obitte N. C., Ofokansi K. C., Nzekwe I. T., Esimone C. O., Okoye I. E. (2011). Self-nanoemulsifying drug delivery systems based on melon oil and its admixture with a homolipid from Bos indicus for the delivery of indomethacin. *Tropical Journal of Pharmaceutical Research*.

[19] Attama A. A., Ezeabasili S. I., Adikwu M. U. (2000). In vitro evaluation of salicylic acid suppositories formulated from palm kernel oil/goat fat mixture. *Journal of Pharmaceutical Research and Development*.

[20] Association of Official Analytical Chemists (AOAC) (1990). *Official Method of Analysis*.

[21] Elnaggar Y. S. R., El-Massik M. A., Abdallah O. Y. (2009). Self-nanoemulsifying drug delivery systems of tamoxifen citrate: design and optimization. *International Journal of Pharmaceutics*.

[22] Taha E. I. (2009). Development and characterization of new indomethacin self-nanoemulsifying formulations. *Scientia Pharmaceutica*.

[24] Batherly P. M. Composition with sustained release of active principle capable of forming micro-emulsion.

[25] Porter C. J. H., Trevaskis N. L., Charman W. N. (2007). Lipids and lipid-based formulations: optimizing the oral delivery of lipophilic drugs. *Nature Reviews Drug Discovery*.

[26] Ofokansi K. C., Chukwu K. I., Ugwuanyi S. I. (2009). The use of liquid self-microemulsifying drug delivery systems based on peanut oil/tween 80 in the delivery of griseofulvin. *Drug Development and Industrial Pharmacy*.

[27] Nielsen F. S., Petersen K. B., Müllertz A. (2008). Bioavailability of probucol from lipid and surfactant based formulations in minipigs: influence of droplet size and dietary state. *European Journal of Pharmaceutics and Biopharmaceutics*.

[28] Obitte N. C., Ezeiruaku H., Onyishi V. I. (2008). Preliminary studies on two vegetable oil based self emulsifying drug delivery system (SEDDS) for the delivery of metronidazole, a poorly water soluble drug. *Journal of Applied Sciences*.

[29] Attama A. A., Nzekwe I. T., Nnamani P. O., Adikwu M. U., Onugu C. O. (2003). The use of solid self-emulsifying systems in the delivery of diclofenac. *International Journal of Pharmaceutics*.

[30] Odeberg J. M., Kaufmann P., Kroon K.-G., Höglund P. (2003). Lipid drug delivery and rational formulation design for lipophilic drugs with low oral bioavailability, applied to cyclosporine. *European Journal of Pharmaceutical Sciences*.

[31] Porter C. J. H., Charman W. N. (2001). In vitro assessment of oral lipid based formulations. *Advanced Drug Delivery Reviews*.

[32] Devani M., Ashford M., Craig D. Q. M. (2004). The emulsification and solubilisation properties of polyglycolysed oils in self-emulsifying formulations. *Journal of Pharmacy and Pharmacology*.

[33] Tang J.-L., Sun J., He Z.-G. (2007). Self-emulsifying drug delivery systems: strategy for improving oral delivery of poorly soluble drugs. *Current Drug Therapy*.

[34] Jeevana J. B., Sreelakshmi K. (2011). Design and evaluation of self-nanoemulsifying drug delivery system of flutamide. *Journal of Young Pharmacists*.

[35] Hörter D., Dressman J. B. (2001). Influence of physicochemical properties on dissolution of drugs in the gastrointestinal tract. *Advanced Drug Delivery Reviews*.

[36] Patil P., Praradkar A. (2006). Porum polystyrene beads as carriers for self-emulsifying system containing loratadine. *Association of American Pharmaceutical Scientists and Pharmaceutical Science Technologists*.

